# Influence of simulated vs. satellite-based burned areas on modelled terrestrial carbon fluxes

**DOI:** 10.1186/s13021-025-00366-5

**Published:** 2026-01-08

**Authors:** Tiago Ermitão, Célia Gouveia, Ana Russo, Chantelle Burton, Evgenii Churiulin, Jefferson Gonçalves de Souza, Michael O’ Sullivan, Philippe Ciais, Sönke Zaehle, Stephen Sitch, Wei Li, Yidi Xu, Ana Bastos

**Affiliations:** 1 Faculty of Sciences, Dom Luiz Institute, University of Lisbon, Lisbon, Portugal; 2 Portuguese Sea and Atmosphere Institute, Lisbon, Portugal; 3 CEF - Forest Research Centre, Associate Laboratory TERRA, School of Agriculture, University of Lisbon, Lisbon, Portugal; 4 MetOffice Hadley Centre, Exeter, UK; 5 Institute for Earth System Science and Remote Sensing, Leipzig University, 04103 Leipzig, Germany; 6 Department of Biogeochemical Integration, Max Planck Institute for Biogeochemistry, 07745 Jena, Germany; 7 Institute of Meteorology and Climate Research Troposphere Research (IMKTRO), Karlsruhe Institute of Technology (KIT), Karlsruhe, Germany; 8 Faculty of Environment, Science and Economy, University of Exeter, Exeter, UK; 9 Laboratoire des Sciences du Climat Et de L Environnement, University Paris-Saclay CEA CNRS, 91191 Gif-Sur-Yvette, France; 10 Department of Earth System Science, Tsinghua University, Beijing, 100084 China

**Keywords:** DGVMs, Satellite-based data, FIRECCI51, Global Carbon Stocks, Burned Area, Model Benchmarking, RECCAP2

## Abstract

**Background:**

The Global Carbon Project provides annual updates on anthropogenic and natural components of the Global Carbon Budget. Dynamic Global Vegetation Models (DGVMs) contribute to these estimates and are used to simulate the evolution of terrestrial carbon sinks. However, DGVMs are known to poorly represent disturbances such as fire, leading to uncertainties in estimates of mean, interannual variability (IAV), and trends in land carbon fluxes. To address this issue, we propose a hybrid-process-based assessmentby constraining three DGVMs (OCN, JULES-INFERNO, and ORCHIDEE-MICT) with remotely-sensed burned areas from ESA CCI (FIRECCI51) and climate data from ERA5 reanalysis. We aim to improve the representation of the spatio-temporal variability of regional carbon budgets, namely fire emissions, above-ground biomass carbon (AGC), and vegetation-related variables—leaf area index (LAI) and gross primary productivity (GPP).

**Results:**

Prescribing burned area (BA) in DGVMs reveals contrasting patterns between prognostic (model simulations) and diagnostic (simulations with prescribed BA) runs. As prognostic tends to overestimate BA, particularly across tropical and high-latitude regions, diagnostic simulations correct this issue, by reducing bias and improving the IAV and the agreement with satellite-based datasets of BA and fire emissions in these regions. Moreover, enhanced IAV of AGC is simulated by diagnostic runs, essentially due to better representation of biomass carbon in the mentioned regions. Although moderate improvements are found in LAI and GPP, as the differences between the two runs are more limited, the improvements between prognostic and diagnostic are more evident in their IAV, particularly for LAI, rather than on long-term means, indicating that prescribed fire can improve the representation of some variability patterns.

**Conclusions:**

Prescribing remotely-sensed BA in models can lead to a better representation of global BA, fire emissions and AGC, particularly improving the IAV, reducing bias and enhancing the agreement with satellite datasets. The moderate improvements in vegetation-related variables underscore the need to better constrain fire impacts and vegetation dynamics in models, to enhance the simulation of spatio-temporal variability and dynamics of regional-scale vegetation and carbon-related fluxes.

**Supplementary Information:**

The online version contains supplementary material available at 10.1186/s13021-025-00366-5.

## Introduction

The Global Carbon Budget (GCB), integrated into the Global Carbon Project (GCP), provides annual updates of anthropogenic influence on carbon stocks from local to global scales [28, 40, 41]. Global spatially explicit models contribute to GCB by estimating net carbon sources, sinks, and their exchanges over time. However, the net annual carbon balance between the anthropogenic sources and sinks estimated by the process-based models does not always match the measures of atmospheric CO_2_ growth [4, 8, 51]. This imbalance can be largely attributed to limitations in datasets and uncertainties in representing different fluxes, mainly related to deforestation, net ecosystem exchange, changes in land cover, land use and management practices [8, 29, 66, 68].

Despite the identified discrepancies at smaller scales, Dynamic Global Vegetation Models (DGVMs) are key tools to simulate local and global terrestrial carbon budgets. Ongoing efforts to enhance the representation of carbon dynamics allow these models to better attribute changes in those budgets to different processes, such as land-use, land-cover change and management (LULCC), elevated CO_2_ levels, nutrient deposition (e.g., NO_2_), and fires [13, 48, 68]. In this context, the pilot project from the European Space Agency—Climate Change Initiative (ESA CCI), REgional Carbon Cycle Assessment and Processes project phase 2 (hereafter, RECCAP2), integrated in GCP, has been delivering accurate global budgets of carbon dioxide, methane, and nitrogen, and promoting synergies between process-based models and Earth Observation (EO)-based datasets, including satellite data for Greenhouse Gas (GHG) estimations and atmospheric inversions of GHG fluxes [18, 22, 37, 39, 61]. As a result, estimations of net carbon stocks and biomass change have been adjusted and improved, helping to identify and correct the uncertainties already mentioned [10, 21, 49].

Climate extremes and disturbances, particularly fire, remain major sources of uncertainty [30]. Poor representations of disturbances and models’ ability to realistically simulate the impacts of fire and following recovery further affects the estimations of carbon uptake by the regrowing vegetation in the following years [43, 49, 54]. Intercomparison projects, such as “Trends and drivers of the regional scale terrestrial sources and sinks of carbon dioxide” (TRENDY), that consists on a set of DGVM simulations using a common protocol and set of driving datasets [60, 61] have been essential in assessing the impacts of droughts and heatwaves on local–regional carbon budgets [6, 9, 42, 57], as well as in evaluating burned areas and fire emissions [5, 16, 17, 24]. However, these studies consistently show that models still struggle to simulate fire seasons length, fuel build-up—especially in drylands and savannas—and, more importantly, represent the interannual variability of burned area [7, 30]. Fire-enabled DGVMs typically represent fire as an emergent process from interactions between climate, vegetation and human activity. Burned areas are simulated as a function of ignitions and fire spread, depending on fuel availability, moisture and weather conditions [30, 55]. The combustion of biomass transfers carbon from live and dead pools to the atmosphere (fire emissions), using emissions factors that vary across biomes and fire regimes. Following fire, the regrowth of vegetation is primarily driven by net primary production (NPP), with biomass pools recovering according to prescribed turnover times that represent background disturbance [55]. Although the algorithm may differ across DGVMs, common challenges remain in simulating realistic fire season length, biomass combustion, and post-fire vegetation recovery, therefore leading to discrepancies in estimating fire emissions, carbon losses, and impacts on vegetation dynamics.

Remote-sensing datasets offer opportunities to improve fire representation in DGVMs, and consequently vegetation dynamics and carbon fluxes. In this work, we analyse the influence of uncertainties associated with simulated fire regimes in carbon and vegetation-related Essential Climate Variables (ECVs), by testing the feasibility of a hybrid process-based between DGVMs and EO-based data, where models are constrained by satellite-driven burned area data from ESA CCI product, FIRECCI51 [45] and climate data from ERA5 [32]. By combining prognostic DGVMs with remote-sensing datasets, this approach allows for delivering improved updates of fire emissions (fFire), natural Above-ground Biomass Carbon (AGC), Leaf Area Index (LAI) and Gross Primary Production (GPP), from regional to global scale. By controlling the uncertainties in fire occurrence and extent, we further aim to inform fire-enabled DGVMs and their host Earth System Models (ESMs) on improvements to fire-related algorithms.

## Data and methods

### Forcing data

To prescribe the burned area (BA) in DGVMs, we use the product FIRECCI51 from ESA CCI [45]. FIRECCI51 is a global product, available with spatial resolutions of 250 m and 0.25 degrees and monthly time-step for the period 2001–2020. This dataset combines information from MODIS to generate and detect the global BA by using the thermal anomalies product (MCD14ML) and the near-infrared reflectance product (MOD09Q1). The algorithm first identifies candidate seed pixels and then applies a region-growing technique to aggregate the active fires and the affected surrounding areas. The algorithm of FIRECCI51 allows to achieve similar or better accuracies among different global BA datasets of burned area detection and omission errors, with a better capture of small BA patches particularly over African savannas, but also in the forests of northern boreal regions, which is relevant for the purpose of our analysis [20, 25, 26, 45]. The algorithm, combined with the detailed spatial resolution, provides a more accurate monitoring of global BA, constituting a suitable dataset for our study.

For all simulations, climate forcing data from ERA5 [32] were used, at 0.25º spatial resolution and hourly time-step, initially from 1950 to 2020, later revised to the period 1960–2020 because of identified preliminary issues with precipitation of ERA5 forcing in the 1950s [11]. The atmospheric CO_2_ forcing is based on global-ice core + NOAA annual resolution used in GCB (1960–2020), the LULCC maps are based on LUH2v2h database [19] and the land cover map is kept fixed to 2010 (same forcing as TRENDY-GCB2021, [61]) at the spatial resolution of 0.25º.

### Model simulations protocol

Modeled burned areas, terrestrial carbon fluxes and vegetation-related variables are derived from three different DGVMs: OCN [62], JULES-INFERNO [15, 46], and ORCHIDEE-MICT [71]. Table 1 provides more details about each process-based model.

**Table 1:** Characteristics of the models used in this study

Model	Spatial resolution	Fire model	Prescribed burned area
OCN	0.50º × 0.50º	Thonicke et al., [62]	Natural PFTs
JULES	1.25 º × 1.875º	INFERNO [15, 46]	All PFTs
ORCHIDEE-MICT	0.50º × 0.50º	SPITFIRE [71]	

OCN and ORCHIDEE-MICT outputs are provided at 0.50º latitude × 0.50º longitude of spatial resolution. For JULES, as outputs are provided at 1.25º latitude × 1.875º longitude of spatial resolution, we remap them to the common grid of 0.50º × 0.50º from its coarser resolution, using an area-conservative weighted remapping technique. We find residual differences between the global totals, which are due to the land/ocean mask between the different models and at different resolutions.

In OCN, BA is prescribed only for natural PFTs, whereas in JULES and ORCHIDEE-MICT is prescribed for all PFTs. It is important to note that JULES simulates its own Plant Functional Type (PFT) distribution and thus, any biases in model simulations disturbance will have a feedback on natural vegetation dynamics. In OCN and JULES, nitrogen input datasets are available via the Nitrogen Model Intercomparison Project (NMIP) datasets [64] and data are available until 2014. Hence, we assume that nitrogen input data remained unchanged between 2015 and 2020.

The protocol description for each model’s spin-up and simulations with burned area is shown in Table 2. For the spin-up, climate data, firstly regridded to the common grid of 0.50ºx0.50º, are cycled multiple times from 1960–1969 for OCN and JULES, while for ORCHIDEE-MICT the years are randomly mixed up.

**Table 2:** Model experiments conducted in this study

Simulation	Co_2_	Climate	LUC	Initialise	Purpose
Spin-up Prognostic	Fixed (1960)	Randomize 1960–1969	Fixed (2010) LUH2v2h	-	Initialize carbon pools
S0				End of Spin-up Prognostic	Control
S2 Prognostic	Time-varying 1960–2020	Time-varying 1960-Dec2020			Evaluate BA simulated by models
S2 Diagnostic	Time-varying 2003–2020			31 December 2002 for S2 Prognostic	Compare with observations

In BA simulated by models (hereafter named prognostic runs) fire is simulated with constant CO_2_ concentration and LU maps from 2010 until the carbon pools are in equilibrium. For the simulations with prescribed BA (hereafter named diagnostic runs), BA from FIRECCI51 is used. Since FIRECCI51 dataset covers two decades, and to avoid a long spin-up calculation time, we conduct a first test in which the diagnostic BA simulation is initialised directly from the equilibrium state of the corresponding prognostic simulation. Due to limitations found in the protocol for the first two years of data from FIRECCI51, the diagnostic runs are initialised in 2003.

The land cover map was fixed to 2010, and regridded to 0.50ºx0.50º common grid, and is implemented following the “S2” simulation protocol of TRENDY-GCB2021 [27, 61], so that our simulations include the effects of elevated CO_2_ and climate change but do not account for changes in land cover. Therefore, and since satellite-based BA from FIRECCI51 does not allow distinguishing between human-made and natural fires, we avoid potential double-counting of fire emissions from deforestation and management, and the resulting spatio-temporal patterns of carbon fluxes and biomass changes reflect, therefore, mostly natural variability components, but also include effects of human-driven fires.

To prescribe burned areas from FIRECCI51, we first convert BA per vegetation type to their corresponding plant functional type (PFT) that can be affected by fire in each model (see Table 1). The global BA for diagnostic runs of JULES (JULES_DIAG_) and ORCHIDEE-MICT (ORCHIDEE-MICT_DIAG_) coincides exactly with FIRECCI51 as expected, given that all vegetation types—natural vegetation and cropland fires—are considered, while diagnostic runs of OCN (OCN_DIAG_) only include fires in natural vegetation. Therefore, the global BA in OCN_DIAG_ is expected to be slightly lower than for the other models. Furthermore, OCN distributes burned area fraction by gridcell coverage of the natural PFTs, weighted by an estimate of PFT-specific flammability and fire resistance, resulting in small differences with FIRECCI51. In contrast, JULES and ORCHIDEE-MICT allocate BA in proportion to gridcell PFT coverage.

Following the spin-up, we conduct the model’s prognostic and diagnostic simulations, and then analyse the corresponding fire emissions, above-ground biomass carbon, leaf area index, and gross primary production simulated by the three DGVMs. To assess the performance of the prognostic and diagnostic simulations, we further compare their outputs against multiple satellite-based datasets.

### Satellite-based Essential Climate Variable (ECV) data for model benchmarking

The details of satellite-based ECVs data used in this study for model benchmarking are described in Table 3. We compare the model simulations for BA with two other satellite-based datasets: Global Fire Emissions Database (GFED4.1s, [67]) and FIRECCILT11 (Óton et al., [50]). GFED4.1s includes small fires, combining satellite information on fire activity and vegetation productivity to estimate monthly burned area at 0.50º spatial resolution. The database spans from 1997 to 2023, estimating BA and fFire at a gridded 0.25º × 0.25º. The FIRECCILT11 (hereafter referred to as AVHRR-LTDR) was also developed by ESA CCI for the period 1982–2018, encompassing global monthly composites estimated at 0.05º pixel resolution. This product uses combined spectral information from both Advanced Very High-Resolution Radiometer (AVHRR) sensor produced by the National Oceanic and Atmospheric Administration (NOAA) and Land Long Term Data Record (LTDR) v5, which is produced by the National Aeronautics and Space Administration (NASA). These products are suitable for our study because the time-series is quite long and thus allows for the benchmarking of mean, IAV and trends of burned areas simulated by DGVMs. For fire emissions estimates, alongside GFED4.1s, we compare the model outputs with Global Fire Assimilation System v1.2 (GFAS1.2) from Copernicus Atmosphere Monitoring Service (CAMS) [36]. This product provides fire emissions from 2003 to present with a spatial resolution of 0.10º, relying on satellite observations of fire radiative power (FRP), which is directly related to the biomass combustion rate [69].

**Table 3:** Description of temporal coverage and the native spatial resolution of satellite-based ECV datasets used to compare with the model’s outputs

Variable	Dataset	Temporal coverage	Native spatial resolution	Reference
Burned Area	FIRECCI51	2001–2020	250 m	Lizundia-Loiola et al., [45]
	GFED4.1s	1997–2016	0.25º	van der Werf et al., [67]
	AVHRR-LTDR	1982–2018	0.05º	Óton et al., [50]
Fire Emissions	GFED4.1s	1997–2020	0.25º	van der Werf et al., [67]
	GFAS1.2	2003–2020	0.10º	Kaiser et al., [36]
Above Ground Biomass	ESA CCI	2010 2017–2020	100 m	Santoro et al., [59]
Leaf Area Index	GLOBMAP	1982–2020	0.08º	Liu et al., [44]
	MODIS	2001–2020	500 m	Myneni et al., [47]
Gross Primary Productivity	MODIS	2001–2020	500 m	Running et al., [58]
	FLUXCOM	2001–2020	0.50º	Jung et al., [35]

We further assess the influence of prescribing BA on vegetation and carbon-related ECVs for which global datasets are available: AGC, LAI and GPP.

To evaluate AGC, model outputs are compared with the satellite-based dataset from ESA CCI, the ESA CCI BIOMASS project, version 4.0 Santoro et al., [59]. This dataset provides annual forest above-ground biomass (AGB) density maps for the years 2010, 2017, 2018, 2019 and 2020 with a spatial resolution of 100 m. The product is generated by integrating multiple observations, including ESA’s C-band, JAXA’s L-band Synthetic Aperture RADAR and space borne LIDAR, and using advanced AGB retrieval algorithms of improved allometries. Because DGVMs simulate total living biomass—including both above- and below-ground components—we scale the modelled biomass carbon values so that we consider only the above-ground carbon. Specifically, we use the above-to-below-ground biomass ratios map from Huang et al. [31] that estimates the fraction of total biomass carbon allocated above ground. The distribution of the AGC fraction is closer to 1 in higher latitudes of the Northern Hemisphere and closer to 0.7–0.8 in equatorial and tropical latitudes, as shown in Figure S1. Then, we apply a similar conversion to the ESA CCI AGB dataset. First, we convert the original units, i.e., MgC/ha in kgC/m^2^ by multiplying by a scale of 0.1 and then, to obtain the amount of carbon of the biomass in each pixel, we applied a biomass-to-carbon conversion factor of 0.47. This factor is recommended by the 2006 IPCC Guidelines for National Greenhouse Gas Inventories (see 2006 IPCC guidelines for national greenhouse gas inventories [1]) and has been widely used in empirical studies to convert dry biomass into its carbon equivalent.

LAI quantifies the area of green leaves covering the ground surface [23] and we compare model simulations with two satellite-based datasets: MODIS [47] and GLOBMAP [44]. The MODIS LAI is available from 2001 to present, and the algorithm derives from the spectral information captured in red and near-infrared bands, using a Biome Property Look-Up Table [38] while the GLOBMAP LAI has a temporal coverage from 1982 to 2020 and the algorithm consists of the long-term combination of both AVHRR and MODIS datasets. Both datasets have a temporal resolution of 8-day, with the primary difference being their spatial resolution: MODIS has a native spatial resolution of 0.005º, while GLOBMAP is at 0.08º.

GPP quantifies the ecosystem-scale photosynthetic flux, and model simulations are compared with EO-based GPP estimates from MODIS Running et al., [58] and FLUXCOM [35]. MODIS GPP has been available since 2001 and is derived using a light-use efficiency algorithm that combines satellite-derived absorbed photosynthetically active radiation (APAR), surface meteorological data, and biome-specific radiation conversion efficiency [72]. FLUXCOM GPP, on the other hand, spans from 2001 to 2020 and the product is generated by an upscaling eddy-covariance flux tower measurements using multiple machine learning algorithms trained with meteorological measurements and satellite data, including LAI, Middle Infrared Reflectance (MIR), or the Normalized Difference Vegetation Index (NDVI) [65]. Both datasets provide GPP with an 8-day temporal resolution, but they significantly differ in spatial resolution: MODIS has a native resolution of 0.005º, while FLUXCOM operates at a coarser 0.50º resolution.

All the satellite-based datasets were re-gridded to the common spatial resolution of 0.50º using area-conservative weighted remapping technique.

### Statistical metrics

To assess the effect of BA prescription, we average the values of BA for the common period 2003–2016 among prognostic, diagnostic, and satellite-based datasets, and calculate the differences between model simulations. For fFire, LAI, and GPP, the common period of both DGVMs runs and satellite-based datasets is 2003–2020, and for AGC, the common period of 5 years includes 2010 and 2017–2020 due to the availability of ESA CCI product.

The interannual variability (IAV) is determined based on annual standard deviation applied to prognostic and diagnostic runs for the common period of the variable, as well as the root mean square error (RMSE). Spatial Pearson correlation coefficients between the model’s simulation and respective satellite-based datasets are also determined. It should be noted that for AGC, the temporal range of the ESA CCI dataset is short, but for the reasons previously described, it is the only product suitable for our analysis.

## Results

### Burned areas

**Fig. 1:**
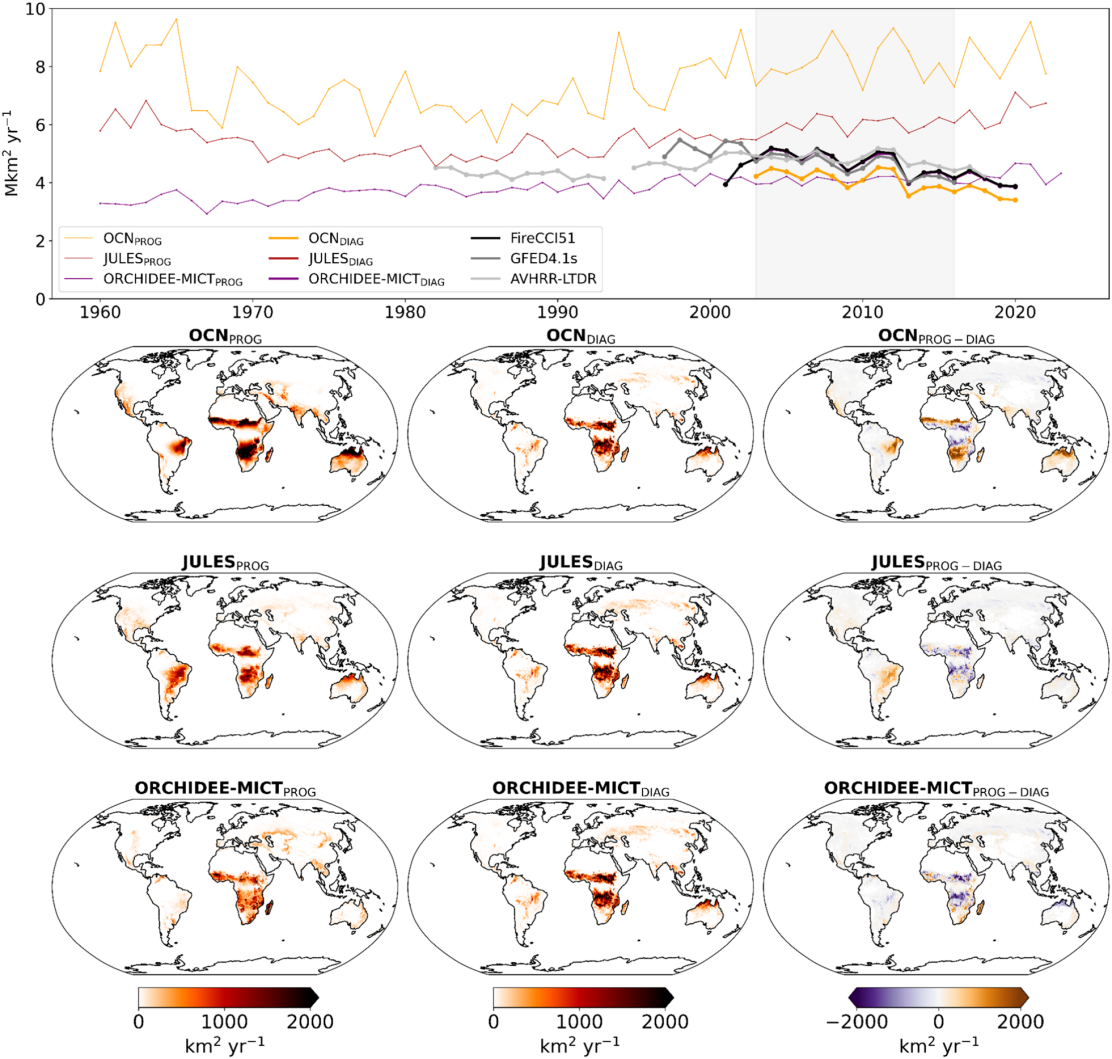
Top panel: Time-series of global annual burned area in Mkm^2^ yr^−1^ simulated by OCN (yellow), JULES (red), and ORCHIDEE-MICT (purple) for the prognostic (thin lines) and diagnostic (bold lines) simulations. The model simulations are compared with satellite-driven datasets, FIRECCI51 (black, overlapping with both JULES_DIAG_ and ORCHIDEE-MICT_DIAG_ simulations), GFED4.1s (dark grey), and AVHRR-LTDR (light grey). The shaded area represents the common period of three DGVMs and satellite-based datasets of BA, 2003–2016; Bottom panel: Spatial patterns of mean burned area in km^2^ yr^−1^ for the common period 2003–2016 simulated by the DGVMs between the prognostic (left panels), diagnostic (central panels), and difference between prognostic and diagnostic simulations (right panels)

We first compare the global annual burned areas simulated by the three DGVMs with prognostic (1960–2020) and diagnostic (2003–2020) runs with BA from GFED4.1s and AVHRR-LTDR (Fig. 1, top panel).

Since the 2000s, both OCN_PROG_ and JULES_PROG_ runs simulate higher global BA than satellite-based datasets (about 8 Mkm^2^ yr^−1^ and about 6 Mkm^2^ yr^−1^, respectively). In comparison, ORCHIDEE-MICT_PROG_ estimates a global BA of about 4 Mkm^2^ yr^−1^, a value already closer to (but lower than) the set of EO-based BA datasets, which generally agree with a global BA of 4.5 Mkm^2^ yr^−1^. The prescription of BA from FIRECCI51 results in lower estimates of global BA compared to prognostic runs, with OCN_DIAG_ simulating about 4 Mkm^2^ yr^−1^ while JULES_DIAG_ and ORCHIDEE_DIAG_ match exactly with FIRECCI51, as expected given the simulation protocol. Moreover, the three prognostic runs simulate an increasing global trend that is at odds with the sustained long-term decline of BA simulated by the three diagnostic runs, as well as the set of satellite-based datasets.

Regional contrasts between prognostic and diagnostic runs of DGVMs are illustrated in Fig. 1, bottom panels. OCN_DIAG_ and JULES_DIAG_ simulate lower BA over regions where the prognostic runs overestimate BA, particularly across tropical and semi-arid regions of the Southern Hemisphere, such as Cerrado in South America and Sahel in Africa. On the other hand, JULES_DIAG_ and ORCHIDEE-MICT_DIAG_ estimate higher BA over subtropical regions of southern Africa and northern Australia. In extratropical zones, especially in boreal regions of North America and Siberia, the three diagnostic runs show an increase of BA, with differences between prognostic and diagnostic of about 500 km^2^ yr^−1^.

**Fig. 2:**
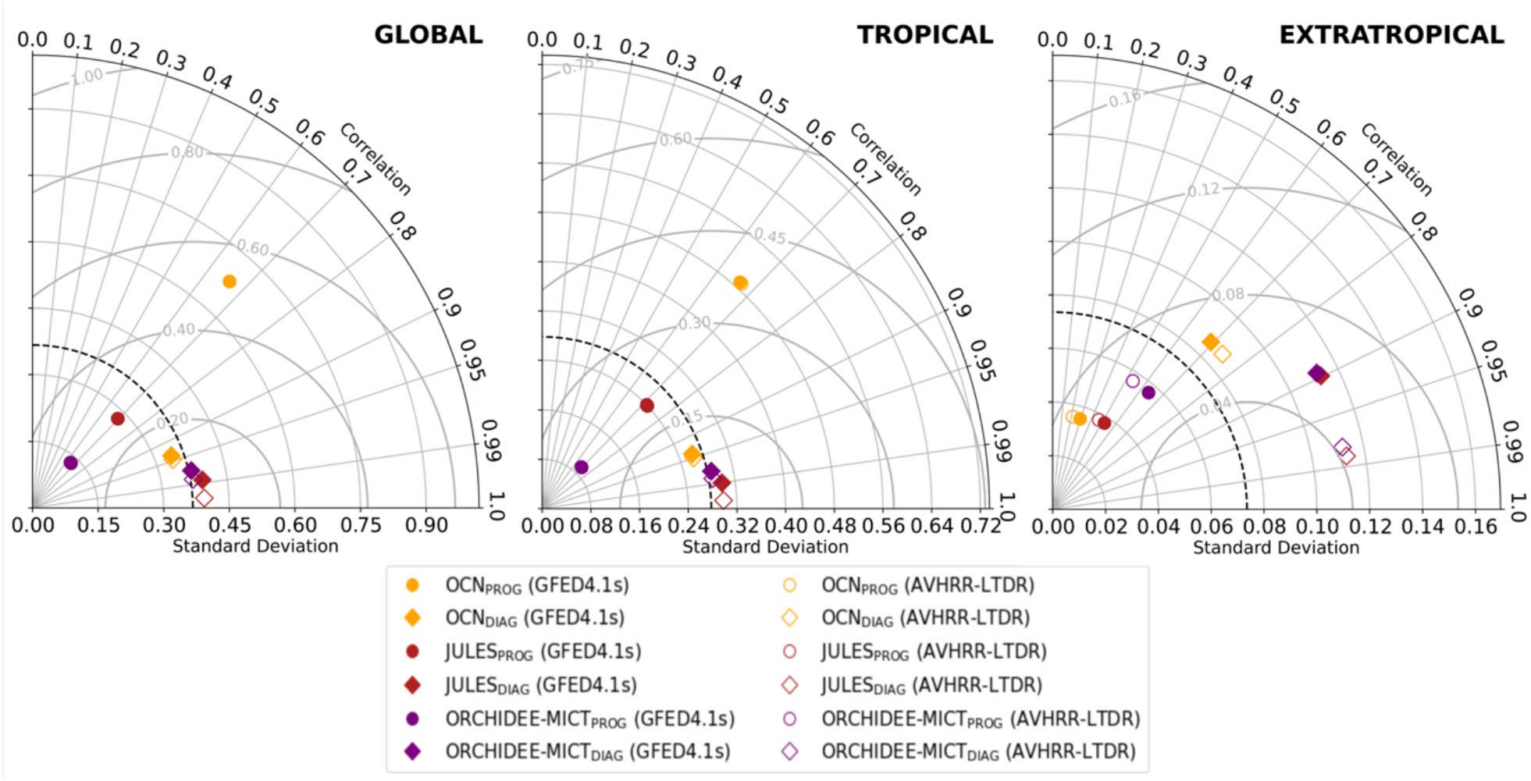
Taylor diagrams of the BA simulations of OCN (yellow), JULES (red), and ORCHIDEE-MICT (purple) for prognostic runs (circles) and diagnostic runs (diamonds), compared with GFED4.1s (coloured) and AVHRR-LTDR (uncolored) in a global scale (left panel), in the tropical band between 20ºN—20ºS (central panel), and in the extratropical band of northern Hemisphere between 45º N—90ºN (right panel). The standard deviation of reference (black dashed contour) corresponds to the mean of all runs. JULES_DIAG_ and ORHIDEE-MICT_DIAG_ overlap. The units of standard deviation and RMSE are Mkm^2^

Important differences are detected in the interannual variability (IAV) of global BA, in correlations with independent EO-based datasets, and in root mean square error (RMSE). These differences are illustrated in the following Taylor diagrams (Fig. 2). Globally (left panel), the IAV decreases from OCN_PROG_ to OCN_DIAG_, contrasting with an increase in global IAV in JULES_DIAG_ and ORCHIDEE-MICT_DIAG_. Additionally, the prescription of BA also enhances the global agreement of model runs with satellite-based datasets, as correlation coefficients shift from about 0.5 (ORCHIDEE-MICT_PROG_) and 0.7 (JULES_PROG_) to approximately 0.95–0.99 in diagnostic simulations, alongside a reduction of RMSE across the three models. These global improvements are mainly driven by alterations in the BA simulation within the tropical band (central panel). Here, JULES_DIAG_ and ORCHIDEE-MICT_DIAG_ reveal an evident increase in IAV, likely due to higher standard deviation in Australia and African tropical forests. In contrast, OCN_DIAG_ shows a reduction in IAV, mainly driven by reductions of standard deviation in Amazonia and northern Australia (see Figure S2 for standard deviation maps). Diagnostic runs in the tropical band exhibit not only correlation coefficients up to 0.95–0.99 but also a significant reduction in RMSE, particularly for OCN, from about 0.60 Mkm^2^ to about 0.20 Mkm^2^. Similar patterns are also evident in extratropical latitudes (right panel), as the three DGVMs estimate an increase in their IAV from prognostic to diagnostic runs, mainly due to higher standard deviation in Siberia (Figure S2). Additionally, for the three models, the correlations increase in the diagnostic runs, especially for JULES_DIAG_ and ORCHIDEE-MICT_DIAG,_ but RMSE does not significantly change.

### Fire emissions

**Fig. 3:**
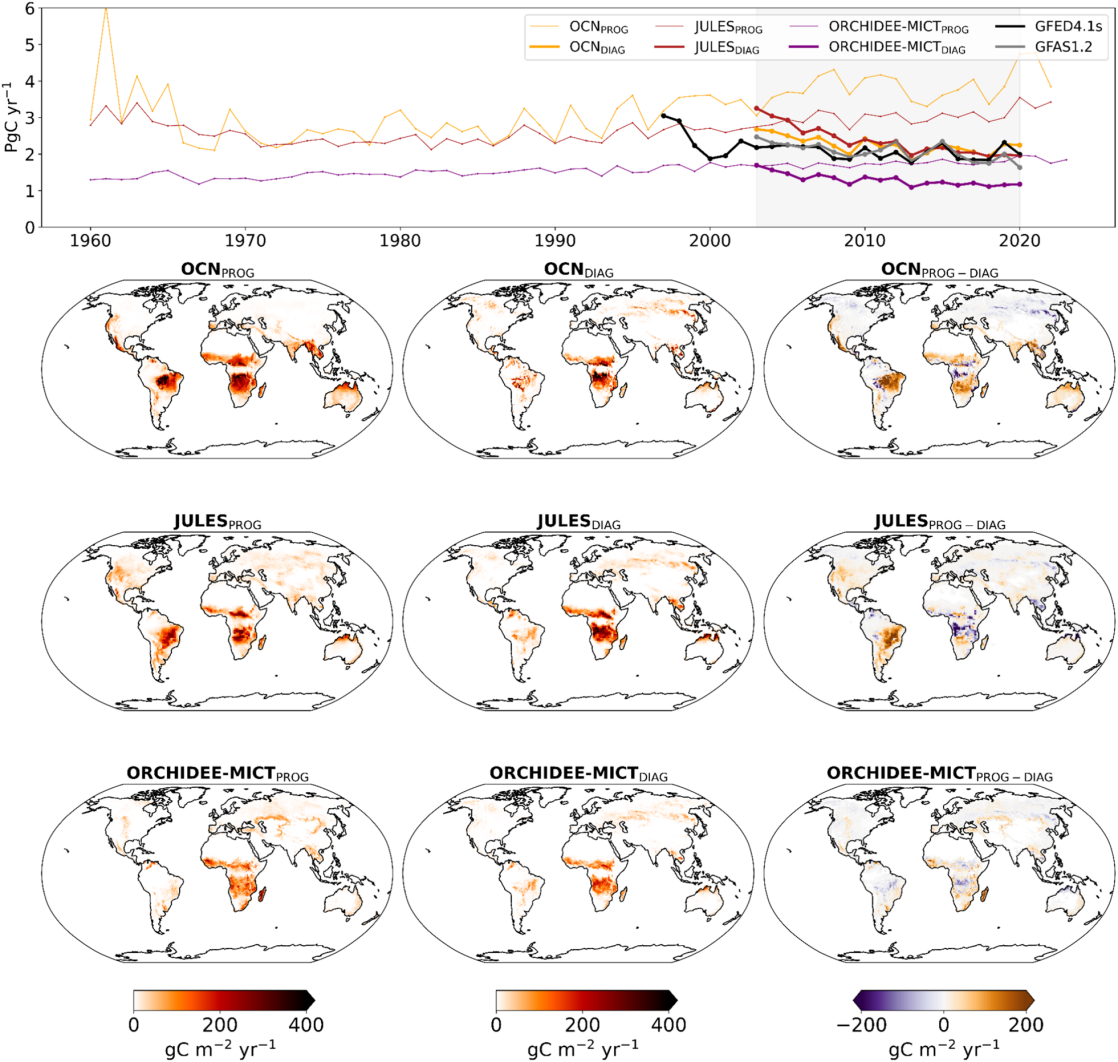
Top panel: Time-series of global annual fFire simulated by OCN (yellow), JULES (red), and ORCHIDEE-MICT (purple) for the prognostic (thin lines) and diagnostic (bold lines) simulations. The model simulations are compared with the fFire satellite-based datasets, GFED4.1s (black) and GFAS1.2 (grey). The shaded area represents the common period of three DGVMs and remotely-sensed datasets of fFire, 2003–2020; Bottom panel: Spatial patterns of mean fFire in gC m^−2^ yr^−1^ for the common period 2003–2020 simulated by the DGVMs between the prognostic (left panels), diagnostic (central panels), and the difference between prognostic and diagnostic simulations (right panels)

In this section, we compare global annual fire carbon emissions simulated by the three DGVMs with both prognostic (1960–2020) and diagnostic (2003–2020) runs against the satellite-based datasets, GFED4.1s and GFAS1.2 (Fig. 3). Both OCN_PROG_ and JULES_PROG_ simulate higher fFire (4 PgC yr^−1^ and 3 PgC yr^−1^, respectively) than the EO-based datasets, GFED4.1s and GFAS1.2, which is strongly associated with an overestimation of BA, as seen in Fig. 1, whereas ORCHIDEE-MICT_PROG_ estimates slightly more than 1 PgC yr^−1^. All three prognostic runs show poor agreement with GFED4.1s and GFAS1.2, which simulate a global fFire of approximately 2–2.5 PgC yr^−1^. The prescription of BA, which led to a reduction of global BA (seen in the previous section) also results in a reduction of global fFire. Although the estimates of both OCN_DIAG_ and JULES_DIAG_ become closer to the satellite-based datasets, ORCHIDEE-MICT_DIAG_ still underestimates fFire relative to the other DGVMs, as well as to GFED4.1s and GFAS1.2. Overall, a decline followed by a recent stabilisation of fFire is observed in the DGVMs and is in good agreement with the satellite-based ECVs.

The maps of Fig. 3, bottom panels, show that spatial differences in mean annual fFire between the prognostic and diagnostic simulations roughly match the differences in simulated BA in each model (see maps of Fig. 1). Despite the decline in global BA, the time-series of diagnostic simulations, particularly the OCN_DIAG_ and JULES_DIAG_, indicate that global fFire has been relatively stable over the past two decades (2003–2020), that may be attributed to reductions in fire emissions over savannas and semi-arid regions, especially in regions such as the Brazilian Cerrado, parts of southern Africa and the Sahel, and northern Australia. Nevertheless, for JULES_DIAG_ and ORCHIDEE-MICT_DIAG_, some regions with higher BA in the diagnostic runs show lower fFire, e.g., in parts of the Sahel and transitional tropical African forests, and in ORCHIDEE-MICT_DIAG_, the reduction of fFire is more pronounced in southern Africa and over parts of the Sahel. In boreal regions of the Northern Hemisphere, the prescription of BA in the three DGVMs leads to a higher fFire, particularly in OCN_DIAG_, which is mainly driven by the increase of simulated BA in these regions.

**Fig. 4:**
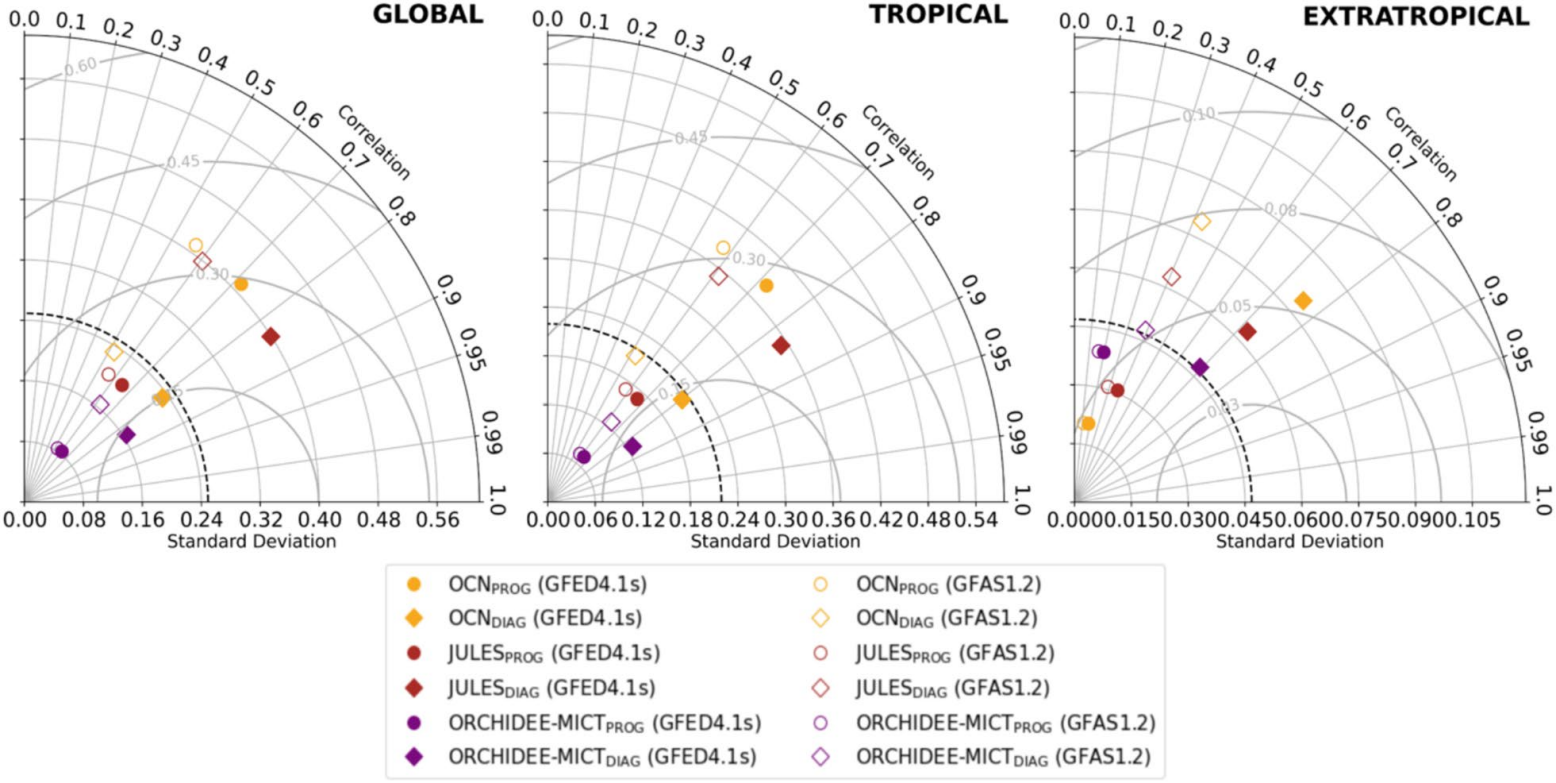
Same as Fig. 2, but for fFire simulations, which are compared with GFED4.1s (coloured) and GFAS1.2 (uncolored). The units of standard deviation and RMSE are PgC

The Taylor diagrams of Fig. 4 highlight important contrasts in the IAV of fFire. Globally (left panel), the IAV simulated by OCN decreases from prognostic to diagnostic runs, associated with the reduction of standard deviation (see maps of Figure S3), and increases from prognostic to diagnostic runs of JULES and ORCHIDEE-MICT. In parallel, more consistent correlation coefficients are observed, particularly with GFED4.1s, which change from approximately 0.60 (ORCHIDEE-MICT_PROG_) and 0.70 (OCN_PROG_ and JULES_PROG_) to about 0.80 in diagnostic runs of the three models. This greater agreement with EO-datasets is due to changes in fFire estimations, particularly in extratropical regions (diagram of the right panel). The higher IAV and RMSE observed in diagnostic runs are intimately related to evident changes in fFire in boreal regions, particularly in Siberia, as previously discussed and also observed in maps of IAV of Figure S3.

We find that the bias reduction (see global maps in Figure S4) is especially pronounced in semi-arid tropical and subtropical regions of Southern America, Africa, and Oceania. The overestimation of fFire by OCN_PROG_ and JULES_PROG_ is reduced, as improved fFire over the regions mentioned before is detected. Nevertheless, high fFire in tropical forests of Africa in JULES_DIAG_ is still noticeable. Regarding ORCHIDEE-MICT, the bias differences are smaller, but some regions also have a switch in the sign of the mean fFire bias, such as Sahel, tropical Africa, and Cerrado. Furthermore, a change in the signal of bias from prognostic to diagnostic runs of fFire in boreal regions, particularly in Eurasia and Siberia, is detected.

After analysing how prescribing BA affects fire emissions, we assess how it affects vegetation-related variables, as these have an influence on changes in available fuel to burn and fire emission factors.

### Biomass and vegetation

Here, we analyse how the prescription of burned area from FIRECCI51 changes the vegetation-related variables of above-ground carbon (AGC), leaf area (LAI) and gross primary production (GPP).

**Fig. 5:**
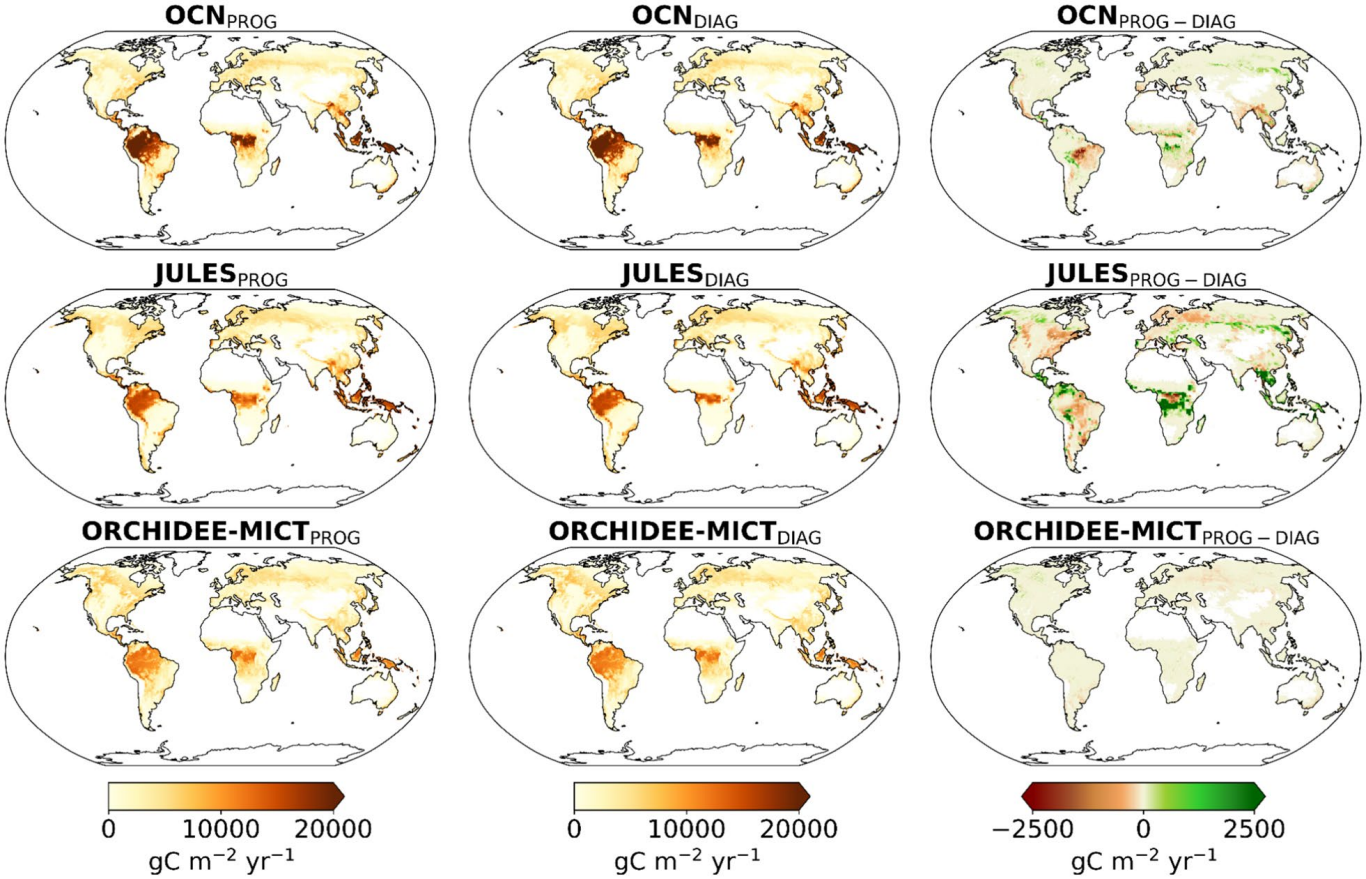
Comparison of spatial patterns of annual mean AGC in gC m^−2^ yr^−1^ for the 5-year period (2010, 2017–2020) simulated by the OCN (yellow), JULES (red), and ORCHIDEE-MICT (purple) between the prognostic (left panels), diagnostic (central panels) and the difference between prognostic and diagnostic (right panels)

Global maps of AGC distribution (Fig. 5) show the three models simulating higher biomass carbon stocks in the forest regions of the tropics, temperate, and boreal regions. OCN_PROG_ simulates the highest AGC densities, particularly over the tropical forests and in transitional areas between the Amazon forest and the Brazilian Cerrado regions. JULES_PROG_ estimates a sharp decrease in the transition between high biomass density in the Amazon forest and low biomass in the semi-arid region of Cerrado. This is because JULES simulates dynamically the natural vegetation distribution and with the inclusion of fire and related feedbacks leads to sharp biome boundaries. In contrast, both OCN_PROG_ and ORCHIDEE-MICT_PROG_, which prescribe natural vegetation distribution, estimate smoother transitions between tropical forests and drylands.

The simulations with prescribed BA result in higher biomass in the transitional regions between the Amazon forest and Cerrado in both OCN_DIAG_ and JULES_DIAG_. This increase in AGC may be driven by lower BA in diagnostic runs in these regions, as previously shown. On the contrary, in transitional areas between humid forests and semi-arid regions in tropical Africa and Asia, as well as over arid regions of South America and parts of southern Africa, OCN_DIAG_ and JULES_DIAG_ estimate lower biomass carbon stocks than in the prognostic runs, consistent with the zonal patterns of higher BA in the diagnostic runs. The same is observed in boreal Eurasia, particularly in Siberia, mainly resulting from the increase of BA in diagnostic runs across these regions. JULES_DIAG_ also reports an increase in biomass stocks in North America and parts of northern Europe. On the other hand, ORCHIDEE-MICT shows small differences in biomass between the two simulations.

**Fig. 6:**
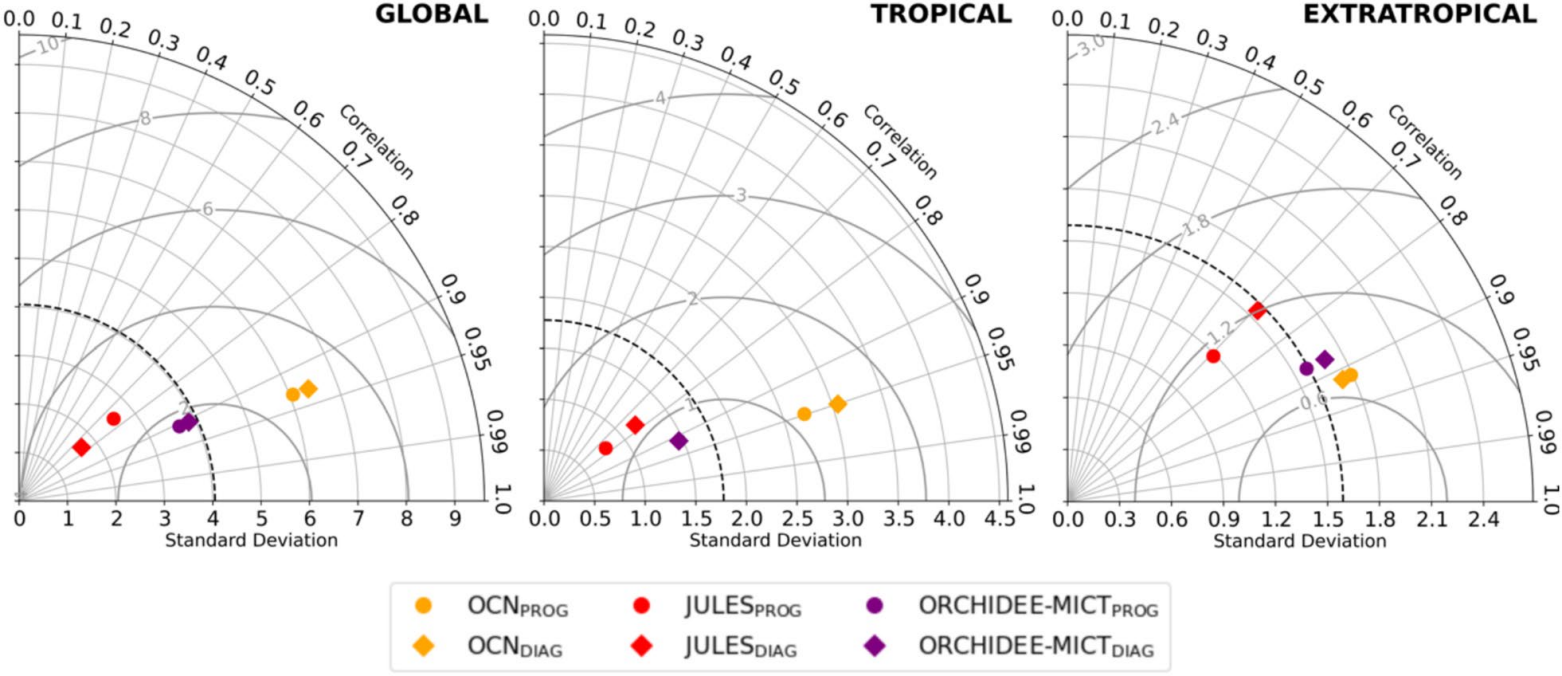
Same as Fig. 2 and 4, but for AGC simulations, which are compared with the satellite-driven dataset from ESA CCI. The AGC units of standard deviation and RMSE are PgC

The Taylor diagrams of Fig. 6 show that, by prescribing BA from FIRECCI51, the IAV of AGC in JULES_DIAG_ increases in both tropical (middle panel) and extratropical (right panel) bands. In turn, OCN_DIAG_ reports an increase of IAV in the tropical band that is partially counterbalanced by the decrease in extratropical latitudes, resulting in small changes in global IAV. In ORCHIDEE-MICT_DIAG_, a small increase of IAV in the extratropical band is detected, which can be linked to the increase of BA simulated in the boreal regions of Siberia. Even though the common period of analysis is short to determine the correlation coefficients between the models and the satellite-based dataset, the Taylor diagrams reveal no meaningful changes in global correlations within the three DGVMs, which range from 0.75 in JULES, 0.90 in ORCHIDEE-MICT and about 0.95 in OCN.

**Fig. 7:**
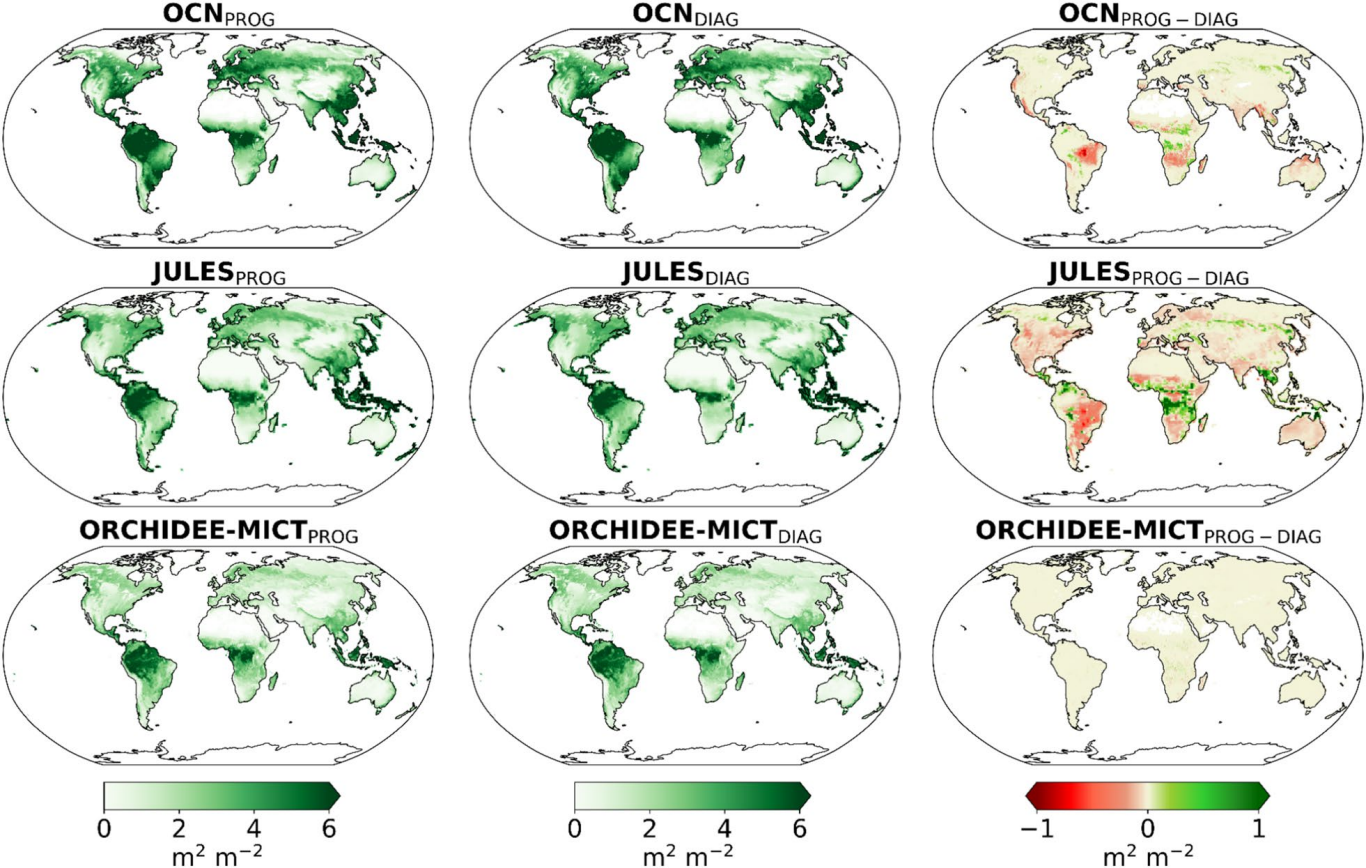
Comparison of spatial patterns of annual mean leaf area index (LAI) for the period 2003–2020 simulated by OCN (yellow), JULES (red), and ORCHIDEE-MICT (purple) between the prognostic (left panels), diagnostic (central panels) and the difference between prognostic and diagnostic (right panels). The unit of LAI is m^2^m^−2^

We then compared how prescribing BA affects LAI and GPP. According to the maps of Fig. 7 (LAI) and Figure S6 (GPP), the regions where the LAI and GPP are the highest, such as the tropical forests of South America, Africa, and Asia, roughly match the areas where the AGC is maximum. Furthermore, LAI and GPP over transitional areas between forest and semi-arid territories, like the Brazilian Cerrado and the African Sahel, have the same spatial pattern as AGC. In these areas, JULES estimates a sharp decrease in LAI, whereas OCN and ORCHIDEE-MICT simulate a smoother transition in values.

The prescription of BA leads to more significant changes in vegetation-related variables in OCN_DIAG_ and JULES_DIAG_. An increase in mean LAI and GPP occurs in regions where a decrease in BA and fFire is verified, such as the Brazilian Cerrado and some territories of southern Africa, while mean LAI and GPP decrease where an enhancement of BA in diagnostic runs are observed, such as in the humid forests and semi-arid regions of tropical Africa and Asia, as well as in the arid regions of South America. These changes are evident in OCN_DIAG_ run and especially in JULES_DIAG_. Moreover, the increase in mean LAI may lead to higher GPP in transitional semi-arid areas of South America and the decrease in LAI leads to lower mean GPP over the tropical forests of Africa and Asia.

Diagnostic simulations also show differences in LAI and GPP across the extratropical Northern Hemisphere, compared to prognostic runs. In OCN_DIAG_ and JULES_DIAG_, mean values decrease at boreal latitudes of Eurasia, particularly in Siberia, where higher BA is detected. By contrast, only JULES_DIAG_ estimates a strong increase in LAI and GPP in North America, which may be attributed to the higher AGC estimation promoted by lower BA and also fFire. In ORCHIDEE-MICT_DIAG_, the global differences in mean LAI and GPP between simulations are almost negligible, although some points in the tropical areas of Africa show positive differences, i.e., a decrease in LAI.

**Fig. 8:**
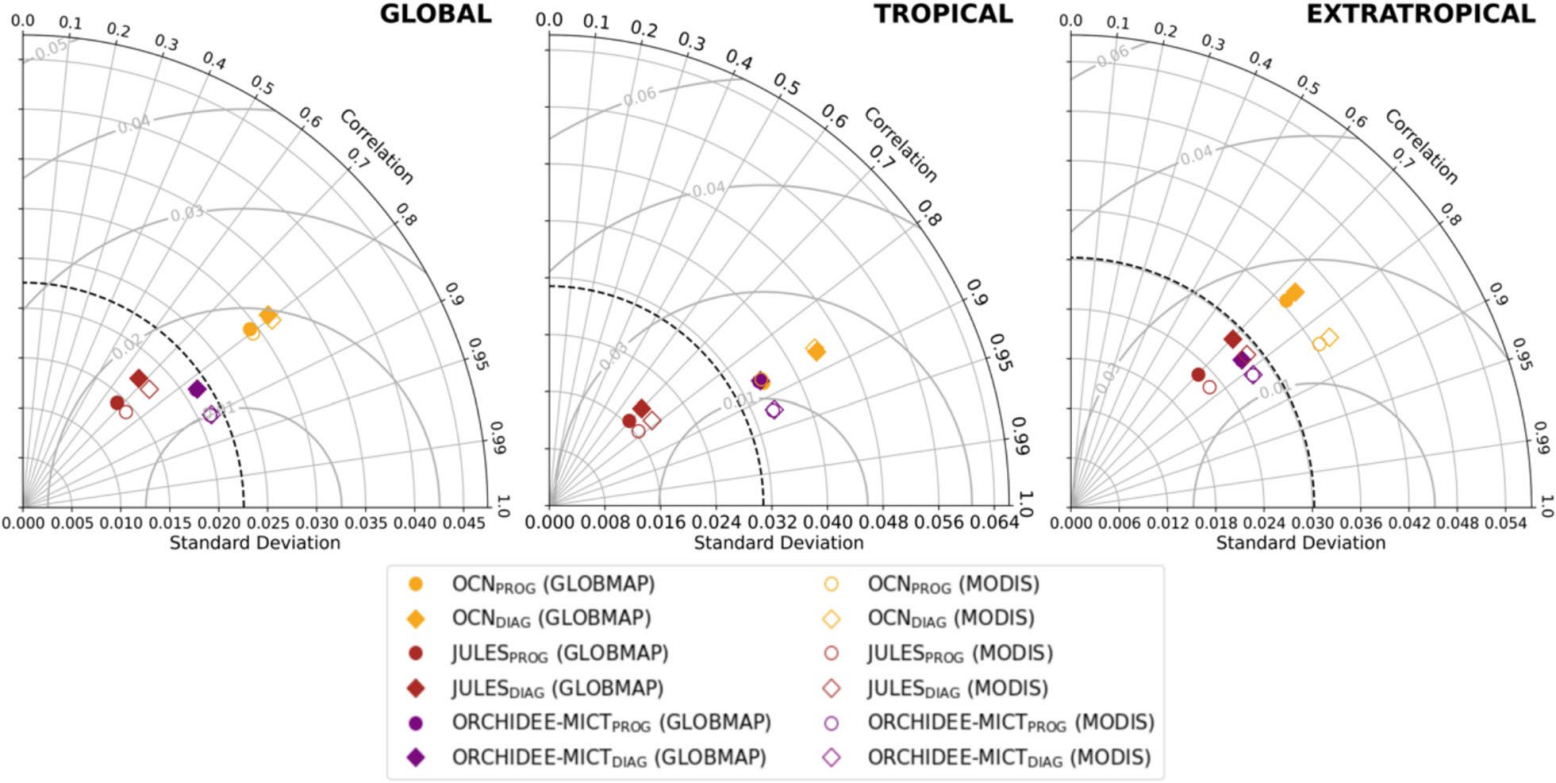
Same as Fig. 2 and 4, but for LAI simulations, which are compared with GLOBMAP (coloured) and MODIS (uncolored). The LAI units of standard deviation and RMSE are m^2^m^−2^

An increase in the IAV of global LAI and GPP is observed from prognostic to diagnostic simulations of OCN, and particularly of JULES (Fig. 8 and Figure S7). This increase is primarily driven by heightened IAV across the extratropical band, especially in the boreal zones of Eurasia and North America. Prognostic simulations already demonstrate strong correlations with satellite-based datasets (GLOBMAP and MODIS for LAI, and FLUXCOM and MODIS for GPP), with coefficients ranging from approximately 0.70 in JULES to 0.80–0.90 in OCN and ORCHIDEE-MICT, showing no significant changes in diagnostic simulations.

## Discussion of results

### Impacts of prescribed burned area on regional fire dynamics

The results with prescribed BA from FIRECCI51 show a general decrease of global BA from prognostic to diagnostic runs in the OCN and JULES. Over the tropical band of the Southern Hemisphere, the decrease of BA extension was more noticeable, due to its decline over African savannahs, associated with changes in fuel driven by hydrological changes, landscape fragmentation, and agricultural activity [3, 33, 34]. At the same time, ORCHIDEE-MICT_DIAG_ reveals a contrasting pattern by simulating higher BA in the same regions. These higher burned area extension might be related to human-related fires considered in FIRECCI51 but not in the “S2” simulations by DGVMs. The model’s simulations run with fixed land cover maps (except JULES, which simulates dynamically natural vegetation), so they do not necessarily capture these environmental and human-driven changes in fuel and landscape connectivity. Therefore, they are likely to have a stronger coupling between fire weather and BA. Nevertheless, it should be noted that recent trends and variability in BA are under scrutiny, given the potential underestimation of global BA products based on coarse resolution remote-sensing and the prevalence of undetected small fires, e.g., in Africa [56]. Furthermore, we detected an evident prognostic-diagnostic contrast in BA between African and South American savannas, particularly in OCN and JULES, which may be associated with biome/region-specific fire response thresholds to moisture and fuel loads, as shown by Alvarado et al., [2], implying that the DGVMs should not use common parameter values for the globe.

The prescription of BA in DGVMs generally increased the IAV and the agreement of modeled fire emissions, which directly depend on BA, with independent satellite-based datasets. According to the results, the differences in global IAV of fFire between simulations showed a consistent and similar pattern to the difference in the variance of BA. Moreover, a greater global agreement with GFED4.1s and GFAS1.2 emissions was noticeable, likely due to improvements in global fFire estimations, especially among high latitudes [12], where fFire has increased over the past years [34]. However, in JULES_DIAG_ and ORCHIDEE-MICT_DIAG_, some regions with higher BA showed lower fFire, e.g., parts of the Sahel and transitional tropical African forests, which might result from lower fuel accumulation under higher fire intensity [56], at least for the JULES model. For ORCHIDEE-MICT_DIAG_, an increase in fFire in some tropical forests, e.g., around the humid tropical forests in Africa, was reported by GFED4.1s, but the DGVMs do not match it. This can be attributed to our simulations not including the effect of land cover and land use change (S2-like runs) while GFED4.1s includes biome-specific emission factors and uses monthly maps of land cover. Results also described that global fFire has been relatively stable in the last decades despite the declines in global BA, agreeing with Zheng et al., [73] findings. This can be linked to the decrease in BA that mostly occurred over African savannas and other semi-arid regions [33, 73]. However, the diagnostic BA output across the three DGVMs likely overestimated the global decline in fFire until around 2010. This could be attributed to the protocol-introduced discontinuity in the fire regime in 2003, as well as to other factors influencing fire emissions that may not be fully represented in DGVMs, such as changes in fuel availability and type, moisture conditions and burning behaviour, that are largely driven by smouldering combustion of woody debris in Amazon and Cerrado regions [24].

### Vegetation-related ECVs response to prescription of burned area

We further show how prescribing BA from satellite-data in DGVMs affects estimates of carbon and vegetation-related variables. The IAV of AGC increases in diagnostic runs over tropics and boreal regions of Eurasia, even though its prognostic runs already show strong agreement with satellite-based AGC data.

In regions where LAI_PROG_ is underestimated, prescribing BA leads to modest changes. OCN strongly overestimates LAI values, especially across tropical bands, likely due to other processes such as nitrogen cycling and elevated CO_2_ effects [63]. In semi-arid regions, the overestimation of mean BA and its IAV by OCN can be explained by the excessive available fuel, as seen in the analysis of LAI and AGC. This reflects the overestimated turnover times in above ground biomass due to the lack of representation of disturbances, both natural and anthropogenic, in DGVMs [70]. We expect that changing BA estimation should modify the fire regimes, and thus impacts the vegetation-related variables. However, the DGVMs, especially OCN, already tend to overestimate LAI, so that it might be closer to its maximum in many regions. Hence, reducing BA does not produce a relevant impact on mean values but rather has a greater influence on IAV. This highlights the importance of capturing the interannual variability, especially in semi-arid ecosystems, which is one of the major contributors to the global carbon cycle IAV [52].

Fire emissions are quite sensitive to changes in available fuel. AGC, LAI and GPP differences suggest that these changes contribute more to differences in fire emission factors than BA differences. This finding contrasts with Poulter et al. [53], who, using only one DGVM (LPJ), found that different EO-based BA datasets resulted in differences in global biomass carbon of up to 300PgC. Although our simulation protocol differs from Poulter et al. [53], we would rather expect stronger discontinuities in the mentioned variables at the onset of the diagnostic simulations, which is not the case. This is likely associated with the fact that we prescribe vegetation cover for two of the three models, limiting the effects of changing fire regimes that are not evidenced in our annual globally integrated time-series and decade-long averages for spatial distribution assessments.

### Implications for model development

Here, we assess how simulations with DGVMs constrained by satellite-data on poorly simulated processes, such as fire, can be used to support regional carbon budget assessments. Prescribing BA from a remotely-sensed product, FIRECCI51, improves the bias, the interannual variability, and the spatial distribution of burned area, fire emissions, and above-ground biomass carbon simulated by DGVMs. This indicates that improving burned area representation by DGVMs is key for better regional carbon budget assessments, particularly in fire-prone regions such as semi-arid tropical regions. However, we found only moderate improvements in the spatio-temporal variability of LAI and GPP when prescribing BA, possibly due to factors such as limitations in the protocol, short common period of analysis among DGVMs and satellite-based datasets, or poor simulation of fire impacts in DGVMs. None of the models distinguishes between burned/non-burned PFT sub-grid tiles. Instead, the biomass is simply reduced after burning, and the fire effects are thereby diluted. This represents a limitation of our study, and may help explain why no significant changes in vegetation-related variables are detected between prognostic and diagnostic runs, particularly in ORCHIDEE-MICT.

We note that FIRECCI51 does not distinguish between natural and anthropogenic fires, which limits our ability to attribute the changes in burned areas and fire carbon emissions, as anthropogenic fires are often seasonal and tied to agricultural activity or deforestation, particularly in tropical regions, while natural fires are more sensitive to climate and fire weather conditions [3, 67]. Additionally, despite the better accuracy on detecting smaller burned areas patches, FIRECCI51 still underrepresents the global cropland burned areas, particularly over regions with high cropland extensions, such as boreal Eurasia or Brazilian Cerrado [26], leading to an underestimation of burned area extensions and fire emissions estimation that should be considered. Future steps may involve extending the period of analysis, as the timeframe used here can be heavily influenced by large-scale atmospheric patterns like ENSO, which induces anomalous and persistent dry conditions in tropical regions [5]. Furthermore, it would also be interesting to run the DGVMs with LULCC to disentangle wildfires from land-management and deforestation fires. This approach could help better constrain the model’s estimates of burned area and fire emissions, particularly in regions affected by land management and ecosystem fragmentation, such as the Sahel.

An important issue that may hinder improvements in vegetation-related variables is the simulation of vegetation regrowth following fire. Current DGVMs typically represent recovery as a simple NPP-driven process with fixed biomass turnover rates [55], which limits their ability to estimate long-term carbon uptake and biomass accumulation. According to Bond et al. [14] and Pugh et al. [54], the often rapid burned vegetation recovery simulated by models is largely due to simplified representations of forest biomass, growth constraints, as well as fire-induced changes in soil properties. Therefore, improving the representation of post-fire recovery remains an important direction for future model development.

We note that many of the satellite-based datasets that are used here as references are also partly modelled, e.g., GFED4.1s fire emissions or MODIS LAI, and they also may have associated uncertainties, especially over regions affected by small fires. Therefore, independent evaluation of fire emissions and vegetation-related variables simulated by the DGVMs should be performed, based on more reliable local data (e.g., eddy-covariance tower fluxes) or based on atmospheric constraints (e.g., CO for fire emissions). Such an exercise is beyond the scope of the current study and project, but can be continued in the future, especially as the pressure towards fast-track assessments of carbon budgets from local to global scales, and from sub-seasonal to multi-annual time scales, increases.

## Conclusions

This study proposes a hybrid process-based between dynamic vegetation models and satellite-driven data, where models are constrained by EO data of burned area from ESA CCI product, FIRECCI51, and climate from ERA5. As disturbances, such as fire, are poorly represented in DGVMs, this framework aims to deliver an improved model’s estimation of burned area, and therefore, a better representation of spatio-temporal variability of regional carbon fluxes, such as fire emissions and above-ground biomass carbon, and vegetation, namely LAI and GPP.

The results show that prescribing BA in DGVMs can improve the simulation of burned area and fire emissions, particularly their interannual variability, and can reduce annual bias relative to satellite-based data. The improvements are evident over tropical and semi-arid regions of Africa and South America, but also over boreal areas of the northern Hemisphere. We note that the effects of prescribing BA on DGVMs are moderate for vegetation-related variables, although improvements are detected in their IAV, essentially over tropical regions.

The overall consistency of our results shows that the synergy between remote-sensing and modelled data can improve the representation of regional and global burned areas and fire emissions, particularly their interannual variability, for different ecosystems. However, the methodology does not fully resolve the mismatches in vegetation responses to fire. Therefore, future efforts should focus on refining the representation of fire impacts and vegetation dynamics, expanding the simulation period, but also enhancing the observation-based constraints for more robust model benchmarking.

## Supplementary Information

Supplementary Material 1

## Data Availability

Model runs from JULES, OCN and ORCHIDEE-MICT have been made available with Forkel et al., (2025) and are available via Zenodo at (https:/doi.org/10.5281/zenodo.14287612).

## Abbreviations

AGB / AGC: Above Ground Biomass / Carbon
APAR: Absorbed Photosynthetically Active Radiation
AVHRR: Advanced Very High-Resolution Radiometer
BA: Burned Area
BK: Bookkeeping Models
CAMS: Copernicus Atmosphere Monitoring Service
CDO: Climate Data Operators
DGVMs: Dynamic Global Vegetation Models
ECVs: Essential Climate Variables
EO: Earth Observation
ESA CCI: European Space Agency—Climate Change Initiative
ESMs: Earth System Models
fFire: Fire Carbon Emissions
FRP: Fire Radiative Power
GCB: Global Carbon Budgets
GCP: Global Carbon Project
GFAS: Global Fire Assimilation System
GFED: Global Fire Emissions Database
GHG: Greenhouse Gas
GPP: Gross Primary Production
IAV: Interannual Variability
IPCC: Intergovernmental Panel on Climate Change
JULES_DIAG_: Diagnostic run of JULES model
JULES_PROG_: Prognostic run of JULES model
LAI: Leaf Area Index
LAI_ANOM_: Annual LAI Anomaly
LTDR: Land Long Term Data Record
LUH2v2h: Land Use Harmonization
LULCC: Land-use, land-cover change and management
MODIS: Moderate-Resolution Imaging Spectroradiometer
NASA: National Aeronautics and Space Administration
NDVI: Normalised Difference Vegetation Index
NMIP: Nitrogen Model Intercomparison Project
NOAA: National Oceanic and Atmospheric Administration
OCN_DIAG_: Diagnostic run of OCN model
OCN_PROG_: Prognostic run of OCN model
ORCHIDEE-MICT_DIAG_: Diagnostic run of ORCHIDEE-MICT model
ORCHIDEE-MICT_PROG_: Prognostic run of ORCHIDEE-MICT model
PFT: Plant Functional Type
RECCAP2: REgional Carbon Cycle Assessment and Processes project phase 2
RMSE: Root Mean Square Error
TRENDY: Trends and drivers of the regional scale terrestrial sources and sinks of carbon dioxide
