# Supplementary material for: Influence of simulated vs. satellite-based burned areas on modelled terrestrial carbon fluxes
Source: Carbon Balance Manag. 2026 Jan 8;21:9. doi: 10.1186/s13021-025-00366-5 (PMC12781732; doi:10.1186/s13021-025-00366-5)
Supplement: Supplementary file 1 — Supplementary Material 1 [file 13021_2025_366_MOESM1_ESM.docx]

**SUPPLEMENTARY MATERIAL**

**
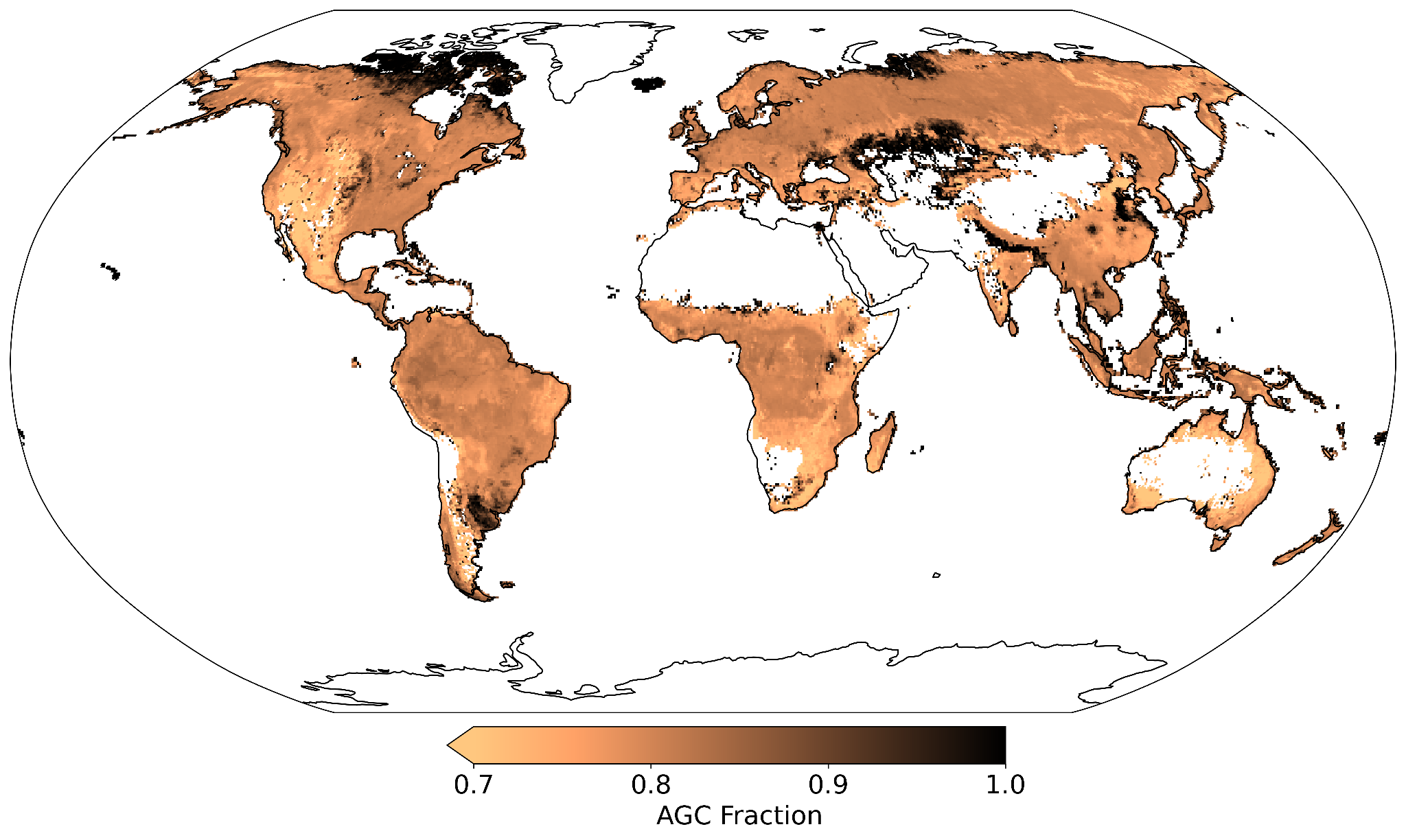
Figure S1.** *AGC fraction of total biomass estimated by Huang et al., (2021).*

**
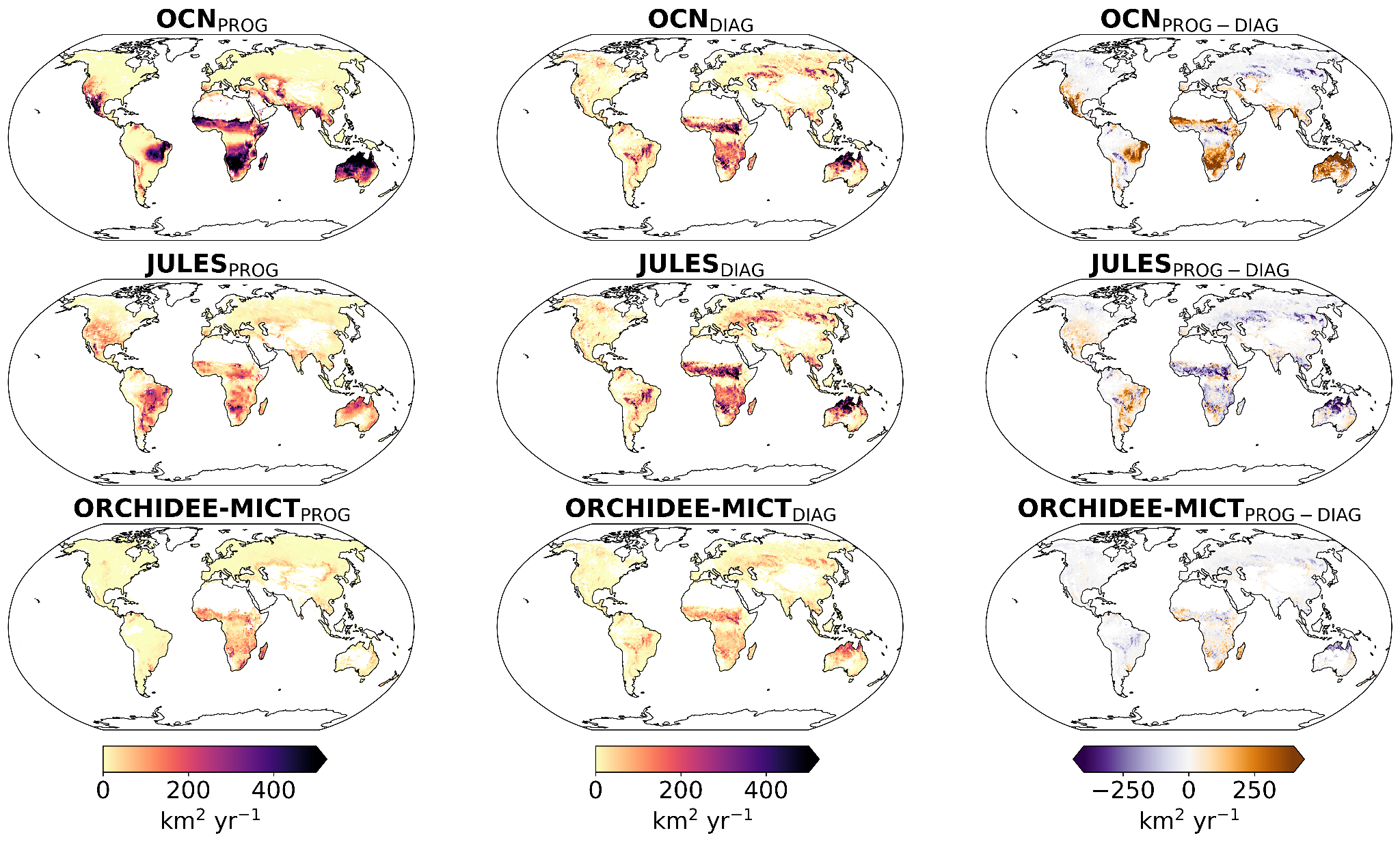
Figure S2.** *Comparison of spatial patterns of BA’s standard deviation (IAV) for the period 2003-2016 simulated by the DGVMs (OCN, top, JULES, middle, ORCHIDEE-MICT, bottom) between the prognostic (left panels), diagnostic (central panels) and the difference between prognostic and diagnostic (right panels).*


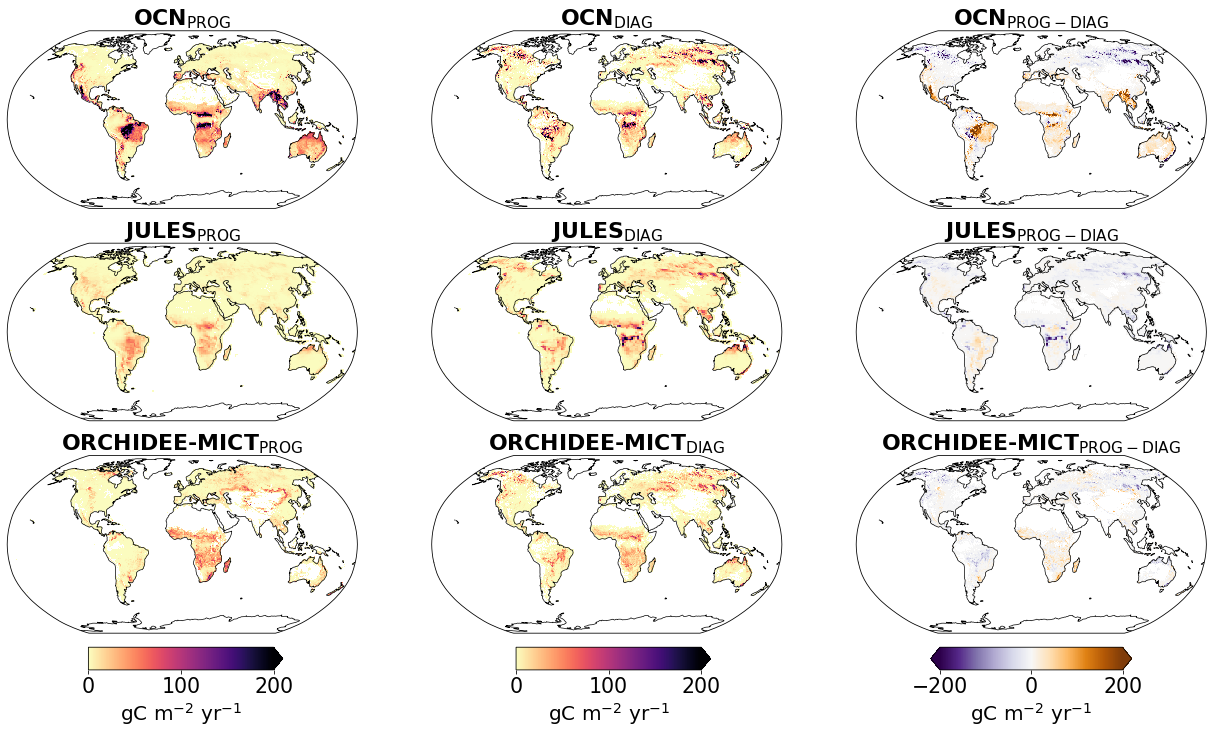
**Figure S3.** *Comparison of spatial patterns of fFire’s standard deviation (IAV) for the period 2003-2020 simulated by the DGVMs (OCN, top, JULES, middle, ORCHIDEE-MICT, bottom) between the prognostic (left panels), diagnostic (central panels) and the difference between prognostic and diagnostic (right panels).*

***
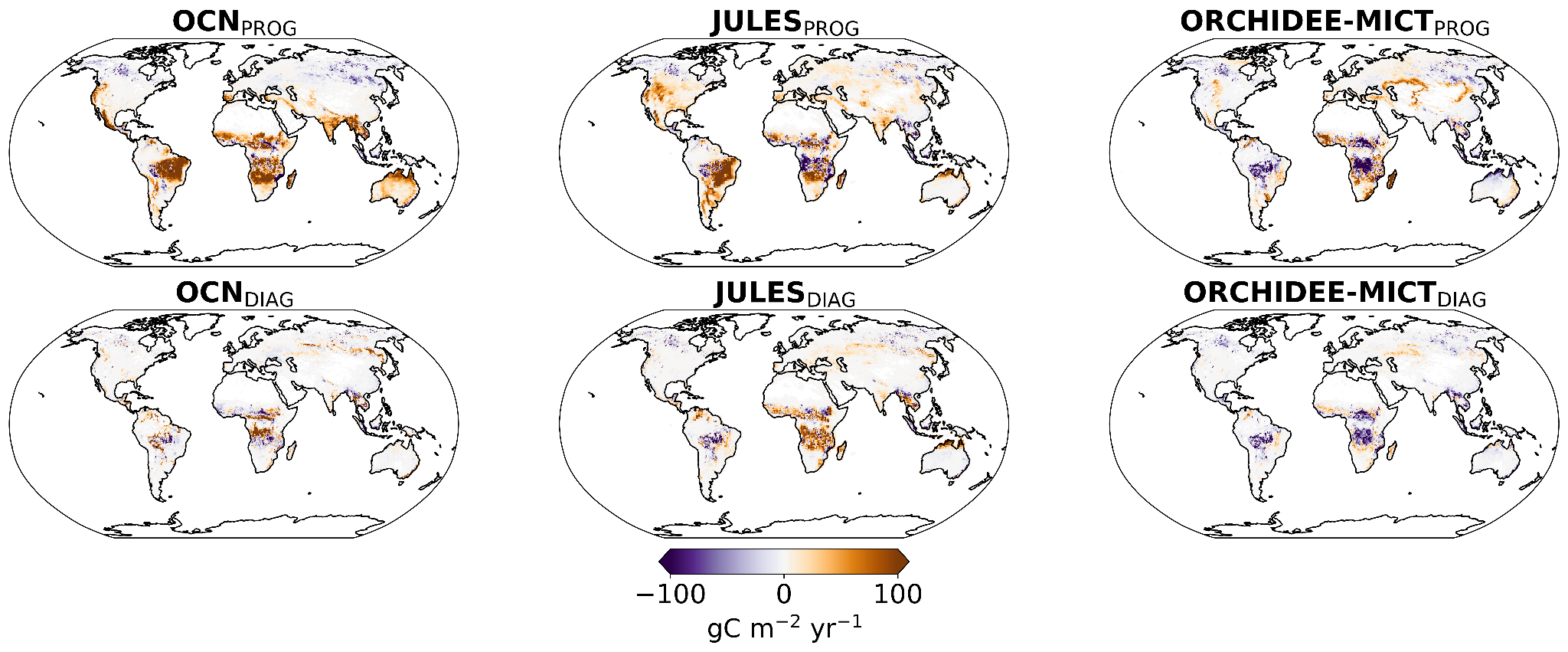
Figure S4.*** *Spatial patterns of the bias of mean annual fFire for the period 2003-2020 between the prognostic (top panels) and diagnostic (bottom panels) simulations of DGVMs and the satellite-based dataset, GFED4.1s.*

**
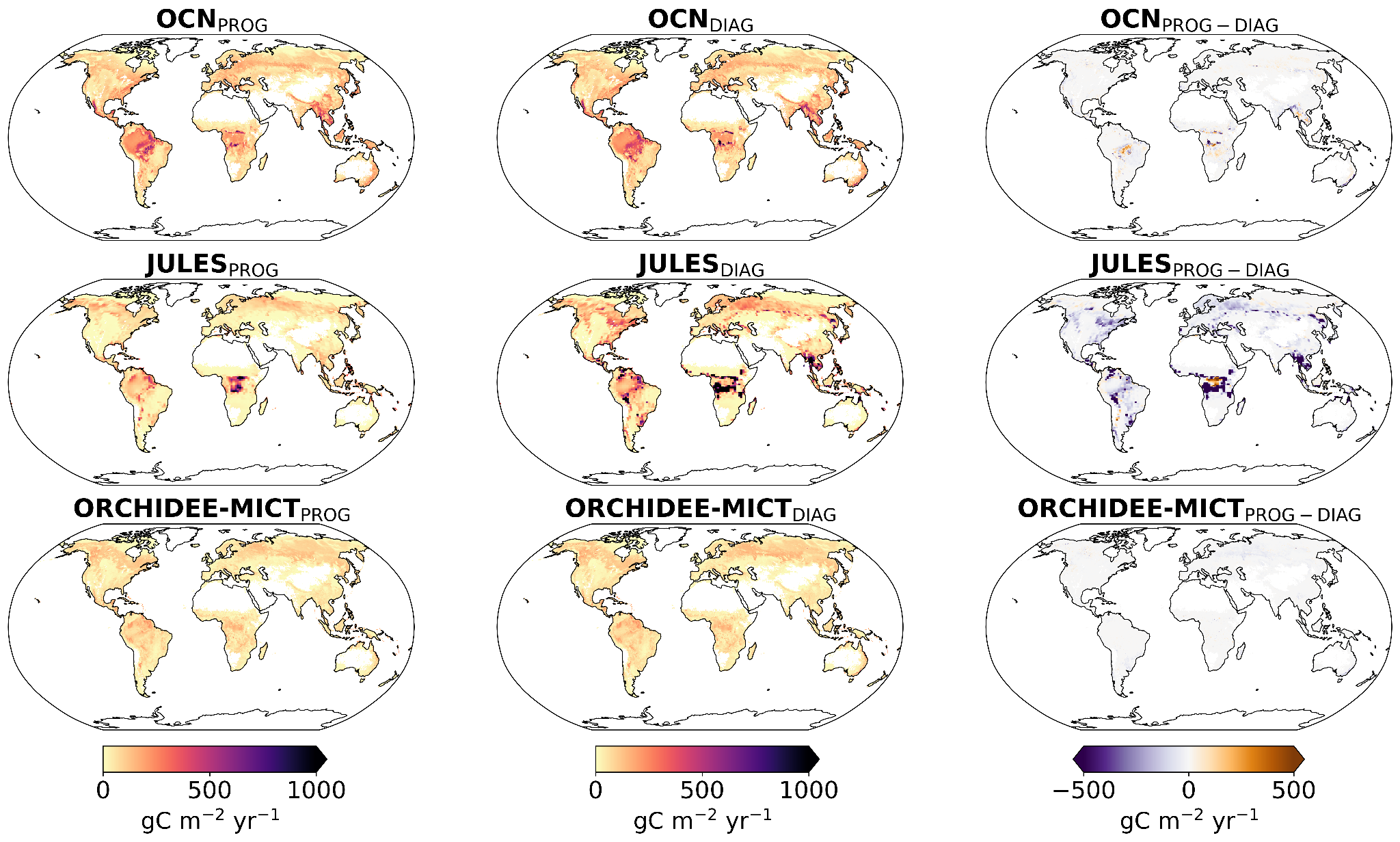
Figure S5.** *Comparison of spatial patterns of AGC standard deviation (IAV) for the 5-year period (2010, 2017-2020) simulated by the DGVMs (OCN, top, JULES, middle, ORCHIDEE-MICT, bottom) between the prognostic (left panels), diagnostic (central panels) and the difference between prognostic and diagnostic (right panels).*

*
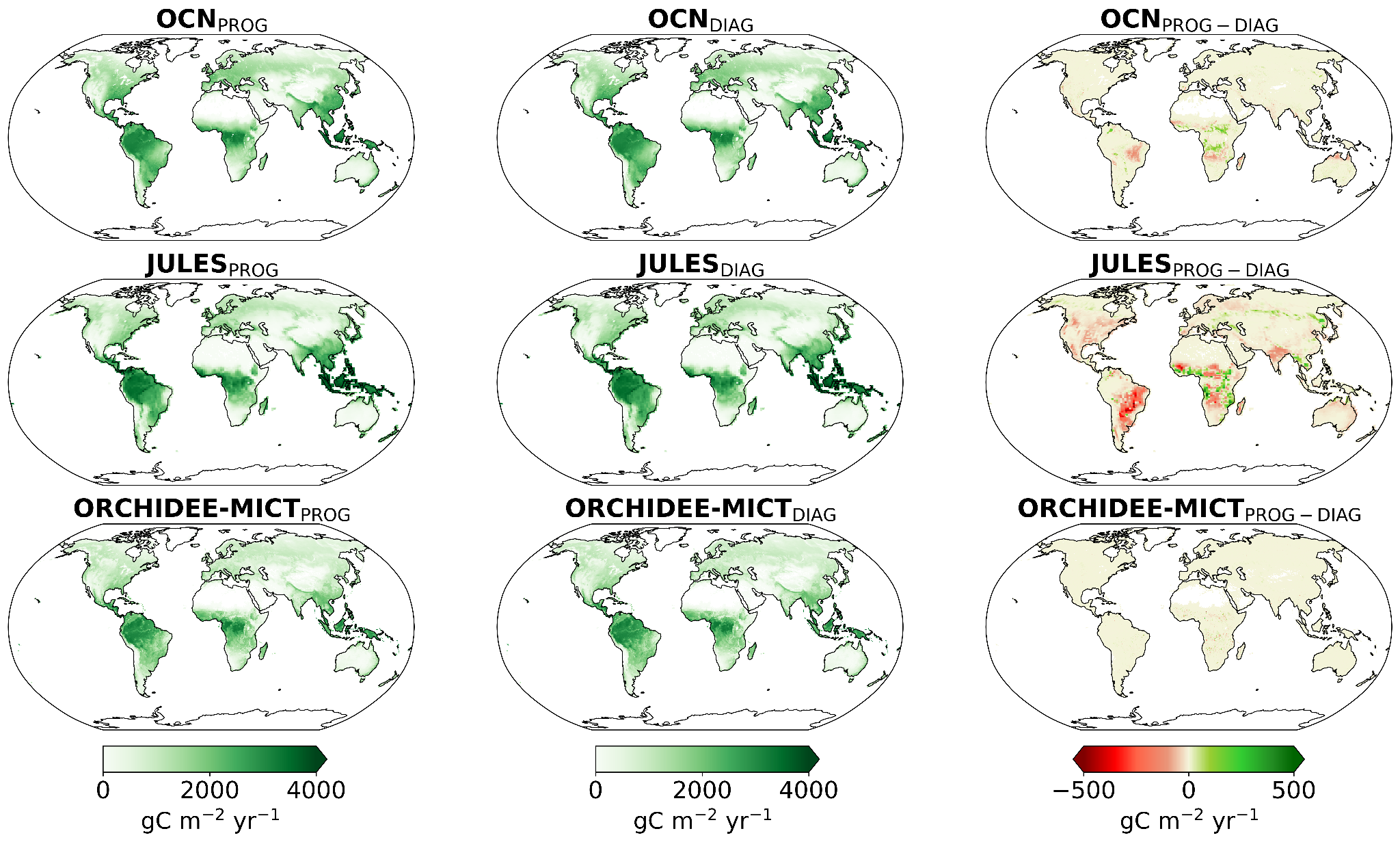
***Figure S6.** *Comparison of spatial patterns of annual mean gross primary productivity (GPP) in gC m^-2^ yr^-1^ for the period 2003-2020 simulated by the DGVMs (OCN, top, JULES, middle, ORCHIDEE-MICT, bottom) between the prognostic (left panels), diagnostic (central panels) and the difference between prognostic and diagnostic (right panels).*

*
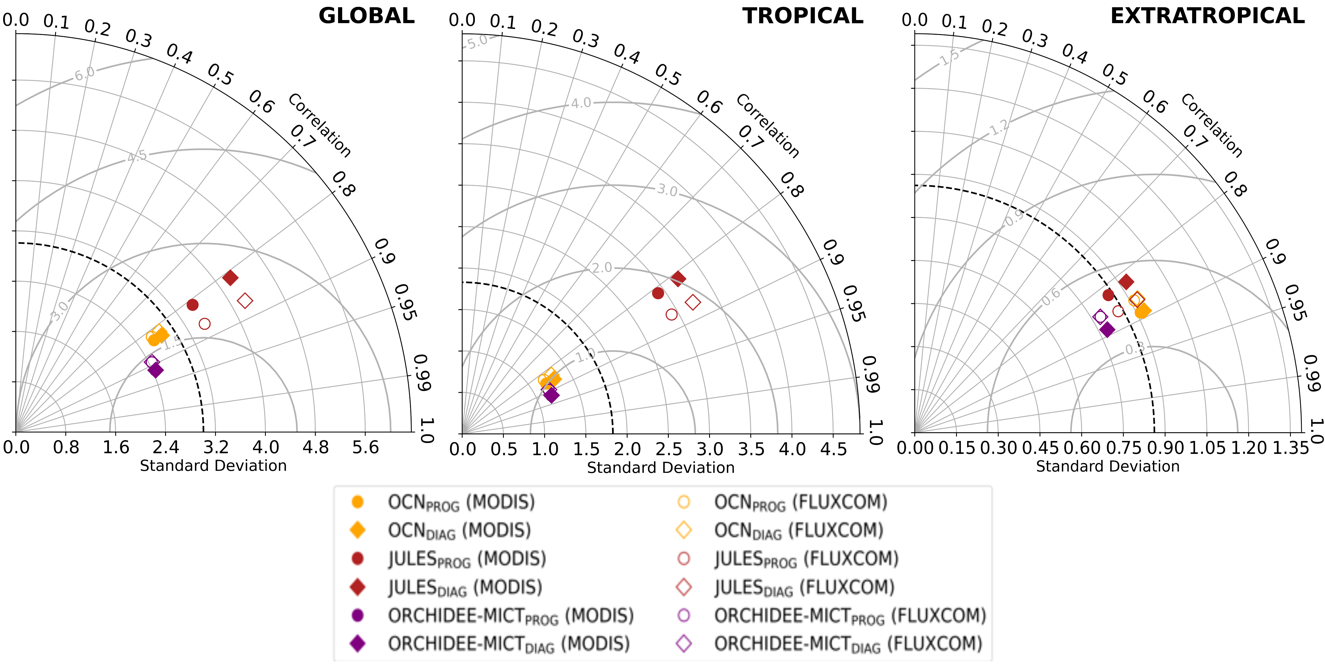
***Figure S7.** *Taylor diagrams of the GPP simulations of the three DGVMs (OCN, yellow, JULES, red, ORCHIDEE-MICT, purple) for prognostic runs (circles) and diagnostic runs (diamonds) compared with MODIS (coloured) and FLUXCOM (uncolored) in a global scale (left panel), in the tropical band (central panel) which range is 20ºN - 20ºS, and in the extratropical band of northern Hemisphere that ranges 45º N - 90ºN (right panel). The standard deviation of reference (black dashed contour) corresponds to the mean of all runs. The units of standard deviation and RMSE are PgC.*
